# ProbeInterface: A Unified Framework for Probe Handling in Extracellular Electrophysiology

**DOI:** 10.3389/fninf.2022.823056

**Published:** 2022-02-15

**Authors:** Samuel Garcia, Julia Sprenger, Tahl Holtzman, Alessio P. Buccino

**Affiliations:** ^1^Centre de Recherche en Neuroscience de Lyon, CNRS, Lyon, France; ^2^Institut de Neurosciences de La Timone, CNRS & Aix-Marseille University, Marseille, France; ^3^Cambridge Neurotech, Cambridge, United Kingdom; ^4^Department of Biosystems Science and Engineering, ETH Zurich, Zurich, Switzerland

**Keywords:** extracellular electrophysiology, open source software (OSS), neural probes, Python (programming language), reproducibility

## Abstract

Recording neuronal activity with penetrating extracellular multi-channel electrode arrays, more commonly known as neural probes, is one of the most widespread approaches to probe neuronal activity. Despite a plethora of available extracellular probe designs, the time-consuming process of mapping of electrode channel order and relative geometries, as required by spike-sorting software is invariably left to the end-user. Consequently, this manual process is prone to mis-mapping mistakes, which in turn lead to undesirable spike-sorting errors and inefficiencies. Here, we introduce ProbeInterface, an open-source project that aims to unify neural probe metadata descriptions by removing the manual step of probe mapping prior to spike-sorting for the analysis of extracellular neural recordings. ProbeInterface is first of all a Python API, which enables users to create and visualize probes and probe groups at any required complexity level. Second, ProbeInterface facilitates the generation of comprehensive wiring description in a reproducible fashion for any specific data-acquisition setup, which usually involves the use of a recording probe, a headstage, adapters, and an acquisition system. Third, we collaborate with probe manufacturers to compile an open library of available probes, which can be downloaded at run time using our Python API. Finally, with ProbeInterface we define a file format for probe handling which includes all necessary information for a FAIR probe description and is compatible with and complementary to other open standards in neuroscience.

## 1. Introduction

Recording neural signals from extracellular electrodes is one of the most widely used techniques to probe neural activity. When electrodes are inserted in the extracellular space of the brain, they pick up the electrical signals generated by neurons, both in the form of action potentials (referred to as spikes) and local field potentials, considered to be the localized sum of extracellular supra- and sub-threshold activity. Since Hubel and Wiesel, in 1957, performed the first extracellular recording using a tungsten microwire (Hubel and Wiesel, 1962), there has been continuous development of neural probe manufacturing technologies aimed at improving usability, spatial resolution, and overall yield of neurons recorded simultaneously. In the 1970's, the first silicon probes where developed, in which metal electrodes are encapsulated in silicon-based shanks (Wise et al., 1970). In the 1980's, researchers started to bundle multiple microwires in *tetrodes* to improve the separation of signals originating from different neurons. In 1990's the Utah array was developed, with 96 electrodes arranged in a grid-pattern; the pioneering technology is still among the most widely used neural probe types for clinical Brain Machine Interface applications. Following this fast-paced development driven by innovations in micro-fabrication techniques, the neuroscience community can now choose from a wide variety of commercially available neural probes tailored to a variety of experimental applications (Hong and Lieber, 2019).

Following the acquisition of an extracellularly recorded signal, it is very common, if not essential for some applications, to extract single neuron activity from the raw signal. This process is referred to as *spike sorting* (Buccino et al., 2020). Recent methods for spike sorting make use of the geometric arrangement of the electrodes on a probe in order to improve the separability of spikes from each of the different single units (Pachitariu et al., 2016; Chung et al., 2017; Jun et al., 2017; Diggelmann et al., 2018; Yger et al., 2018; Lee et al., 2020). For example, when using tetrodes, spike sorting is usually performed tetrode-wise because the distance between different tetrodes precludes recording the same spike on multiple tetrodes. Similarly, when using silicon probes or recent high-density micro-electrode arrays (Frey et al., 2009; Jun et al., 2017), spike sorters can utilize the geometry of the probe to model the spatial location and extent of individual neuron spike signals, thereby improving spike sorting performance.

Currently, however, it is in most cases left to the end user to correctly retrieve the mapping information for their probe from the vendor, to parse the geometric information into file formats that are specific to their chosen spike sorter (e.g., a .prb file for Klusta, Spyking CIRCUS, a .mat file for Kilosort and Ironclust, etc.), and to properly verify that the wiring between each recording electrode and the acquired signal is consistent and correct. This last step can be quite tedious and error-prone as most probes require connection to the data-acquisition system via headstages and / or adapters which result in a re-ordering of the signals.

In order to address these issues and to make probe handling less error-prone and more reproducible, we introduce ProbeInterface, a Python package for unified and standardized probe handling in neuroscience. ProbeInterface is first of all a Python API (Application Programming Interface) that enables users to define, visualize, and use probe definitions for their data analysis. Second, ProbeInterface comes with a probe library that was built in concert with Cambridge Neurotech, which is one of the major vendors of extracellular neural probes. Users can download any available probe model from the ProbeInterface library (https://gin.g-node.org/spikeinterface/probeinterface_library) in a single line of code. Third, ProbeInterface allows users to quickly describe the correct wiring between the probe and the recording device through a series of commonly used adapters and headstages. Finally, ProbeInterface comes with a JSON-based file format that carries all relevant metadata about the probe, including the geometry of the electrode (position, shape, size), the intrinsic channel indices, the size of the silicon shanks, and more.

## 2. Overview of ProbeInterface

**Figure 1:**
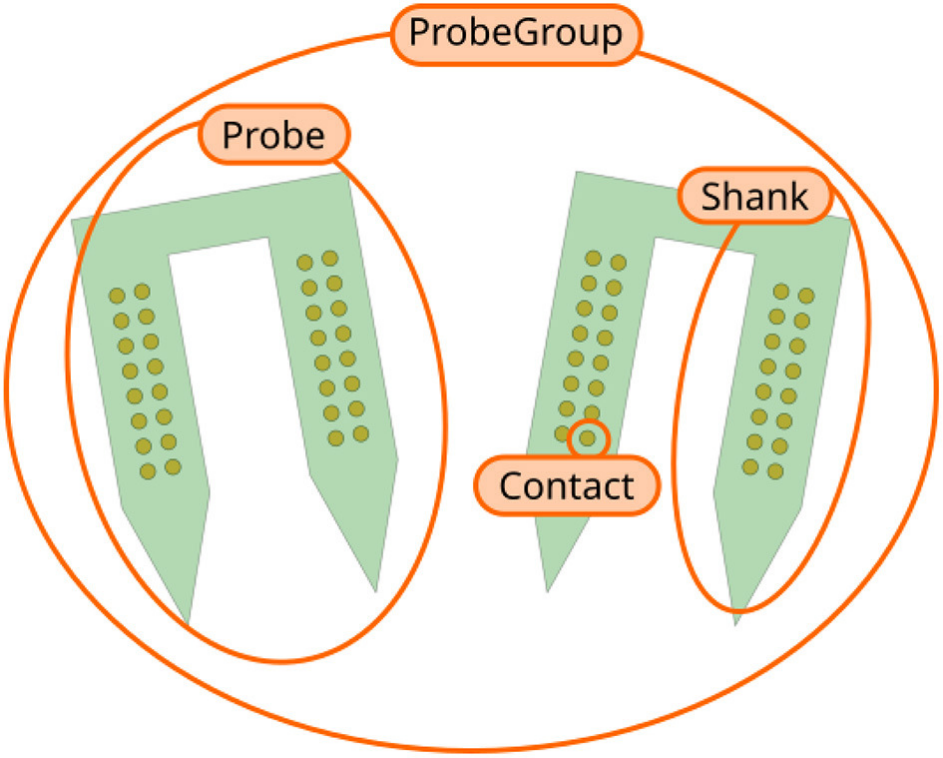
Overview of ProbeInterface objects. Each recording site is a *Contact*. Several contacts make up a *Shank*, which is a region of the probe with multiple contacts. A *Probe* can consist of multiple shanks (two in this figure). Finally, several *Probe* objects can be combined into a *ProbeGroup*.

ProbeInterface describes neural probes in terms of the following core concepts (Figure 1):

- *Contact*: a *Contact* is a recording site with information about its position, shape, size, and additional optional metadata (e.g., material, coating, impedance).
- *Shank*: a *Shank* represents a region of the probe with multiple contacts. As many probes can have multiple shanks, the Shank object adds a shank_id index to each contact.
- *Probe*: a *Probe* is a collection of shanks. Probes further add a unique contact_id to each contact, which represents the internal channel naming provided, for example, by the probe manufacturers. Additionally, *Probe* objects can also provide information about the overall probe shape, which can be used for plotting or modeling purposes.
- *ProbeGroup*: a *ProbeGroup* is a collection of multiple probes used in an experiment connected to the same acquisition device.

These simple concepts allow us to comprehensively describe any collection of probes used in an electrophysiology experiment and to provide essential probe information to data analysis tools in a standardized fashion.

## 3. Getting Started With ProbeInterface

ProbeInterface is a lightweight Python package that can be installed from the Python Package Index (PyPI) (https://pypi.org/project/probeinterface/):


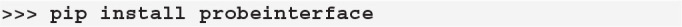


The source code is open and available on GitHub (https://github.com/SpikeInterface/probeinterface) and an extensive documentation with several examples is available on the documentation page (https://probeinterface.readthedocs.io). In this manuscript we are referring to ProbeInterface version 0.2.6. A notebook to generate the figures can be found at https://spikeinterface.github.io/blog/probeinterface-paper-figures/.

In the following sections, we will show some example use cases using ProbeInterface to create ProbeGroup objects from scratch, to retrieve available commercial probes from the ProbeInterface library, and to wire a group of probes to a specific device, also termed channel mapping.

### 3.1. Creating a Probe Configuration From Scratch

Let us first assume that we are using a custom probe design developed by a collaborator. The probe has two shanks and in our experiment we use two of these probes in different brain regions.

We can start by importing the packages we need, including ProbeInterface:


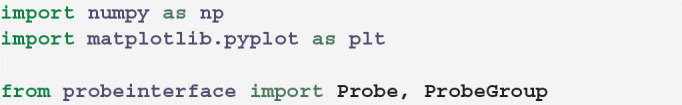


We can next define the position of each contact. In our toy example, let us assume that each shank has two columns of 8 contacts, so that each probe has 32 contacts:


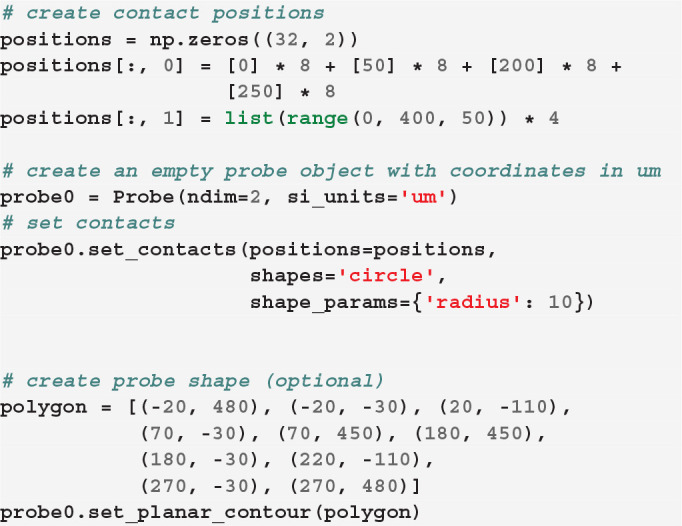


Now that we created our first probe probe0, we can add a second probe with the same design, but at a different location. The two probes need to be aggregated into a probe group:


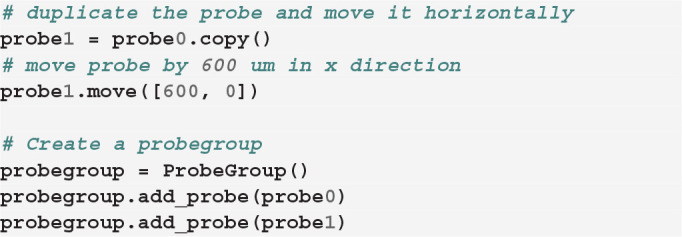


**Figure 2:**
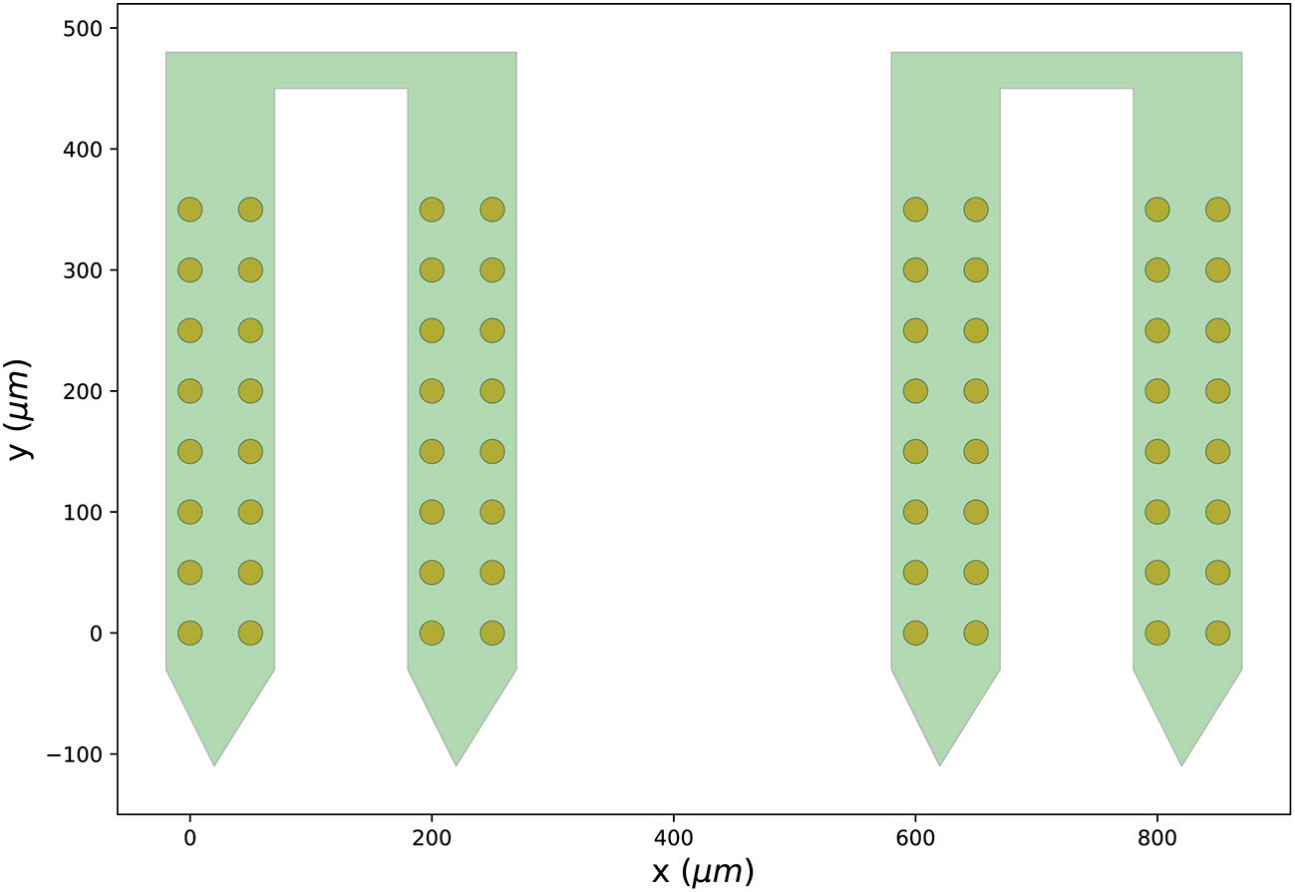
Probe configuration created from scratch. Visualization of the probe group created in Section 3.1. The probe group is made of two identical probes spaced by 600μm in the x direction. Each probe contains 32 channels, split in two shanks with 16 contacts each.

Finally, we can visualize the newly created probe group using the ProbeInterface plotting module (the output figure is Figure 2).


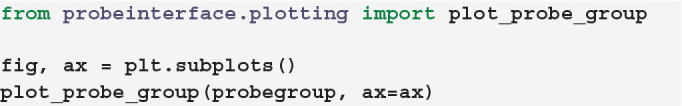


### 3.2. Using the Probe Library

In most cases, neuroscientists use commercially available neural probes. While manufacturers provide the necessary information to use their probes, the probe metadata are typically provided as non-standardized and highly vendor-specific catalogs. During the initial design and implementation of ProbeInterface, we collaborated with one of the major vendors of silicon probes (Cambridge Neurotech, UK) to gather, curate, and distribute most of their available probe designs in a standardized and easy-to-access format. In addition, we are in contact with other vendors (NeuroNexus Technologies, USA, ATLAS Neuroengineering, Belgium) and we have started porting some of their probe designs into ProbeInterface. We have produced a library of probes that is hosted and maintained on the GIN platform (https://gin.g-node.org/spikeinterface/probeinterface_library). From the Python API, users can retrieve any available probes as a ProbeInterface Probe object from the above-mentioned vendors with a single line of code.

In the following example, a Cambridge Neurotech probe (ASSY-156-P-1) and a NeuroNexus probe (A1x32-Poly3-10mm-50-177) are retrieved from the probe library, combined into a probe group, and visualized:


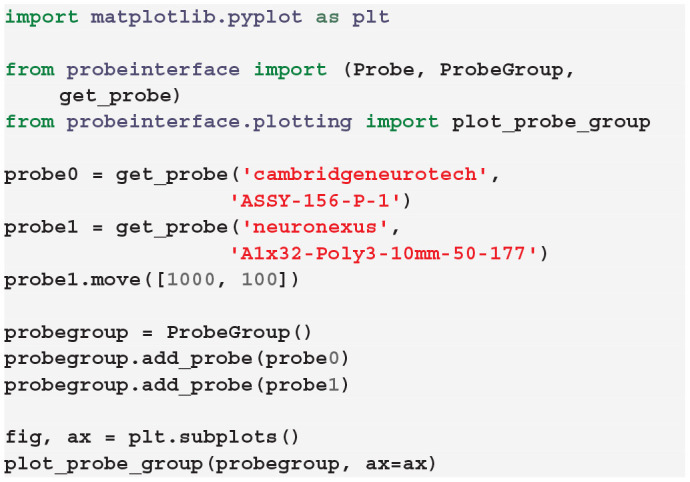


**Figure 3:**
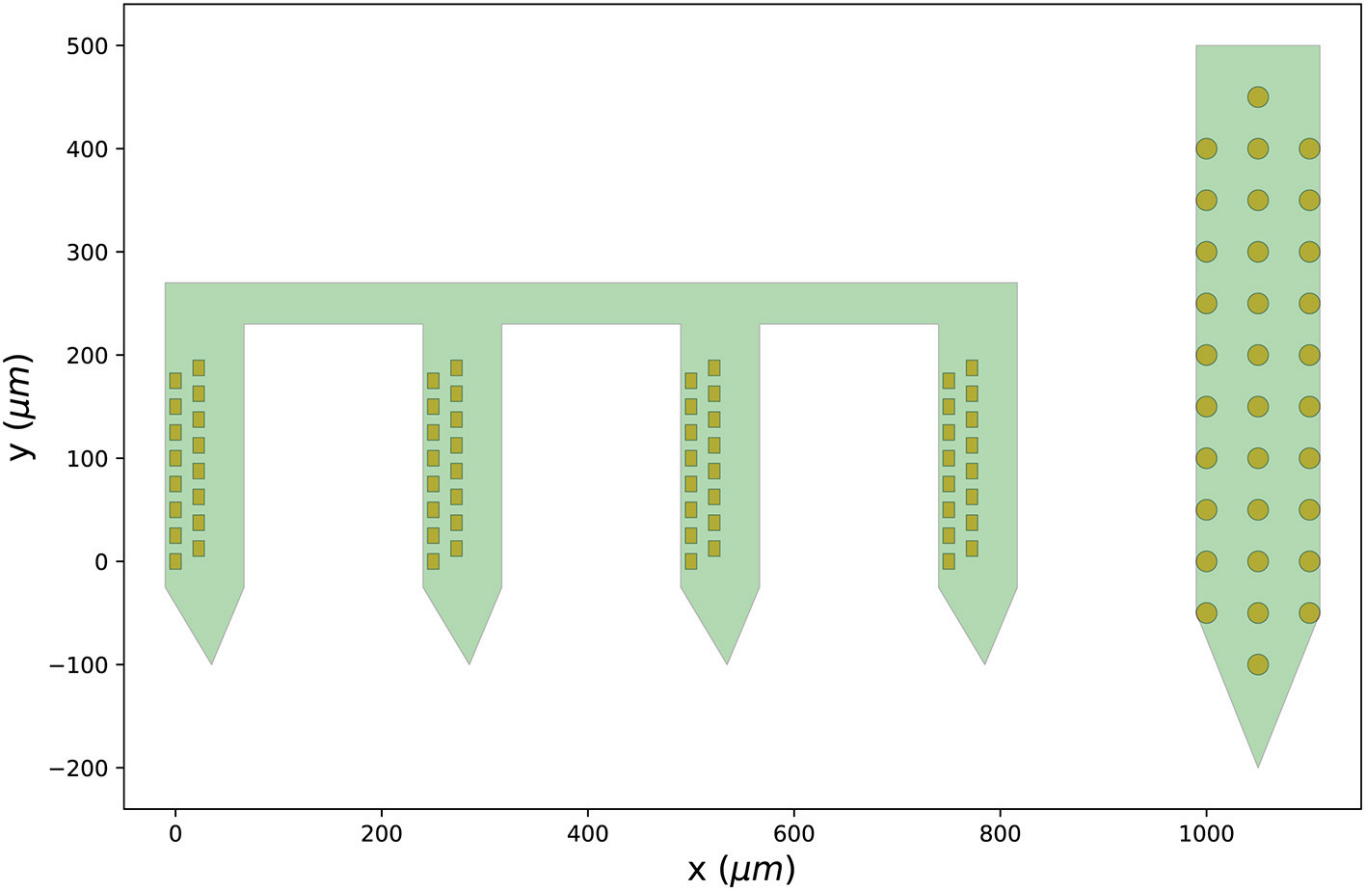
Probes downloaded from the probe library. Visualization of the probe group created in Section 3.2 including a Cambridge Neurotech probe (ASSY-156-P-1 – left) and a NeuroNexus device (A1x32-Poly3-10mm-50-177 – right).

Figure 3 shows the output figure, containing the Cambridge Neurotech probe on the left (64 channels distributed over four shanks with 16 contacts each) and the NeuroNexus probe on the right (32 channels organized in three columns of 10, 12, and 10 contacts). Once probe models are downloaded, they are cached by ProbeInterface and do not need to be downloaded again.

### 3.3. Wiring Probes to an Acquisition Device

The last section showed how to retrieve commonly used probe models as ProbeInterface objects from the probe library. However, there is still a required step in order to map the recorded signals to the probe contacts: *wiring*.

While each contact has a specific contact_id representing the channel identity with respect to the probe, the final ordering of the channels is usually shuffled when probes are connected to an acquisition device *via* headstages and connectors. This is why probes need to be *wired* to a device.

If the channel mapping to the acquisition system for a certain probe and configuration is known, it can be directly connected to the probe configuration. In this example, we first retrieve a Cambridge Neurotech ASSY-156-P-1 probe object (same probe shown in Figure 3— left) and we then *manually* set the device indices using our known mapping.


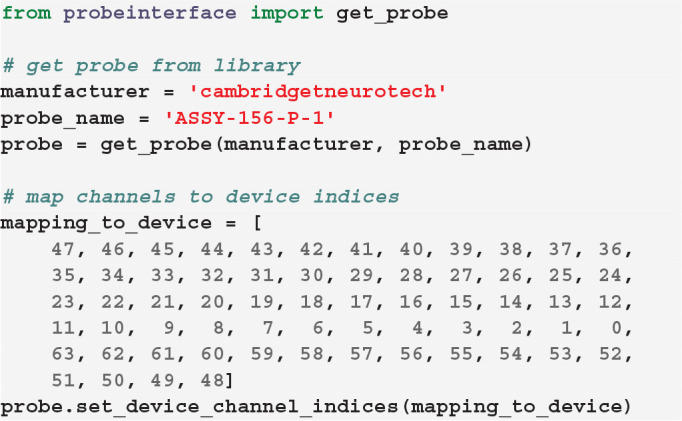


In this case, for example, the first contact of the probe will be mapped to the device index 47, the second to 46, and so on.

Alternatively, we provide a set of available and common *pathways* that allows automatic mapping of probes to connected devices. For example, the ASSY-156-P-1 probe by Cambridge Neurotech usually comes with an “ASSY-156” connector. This is interfaced to an Intan headstage with 64 channels (“RHD2164”) and then connected to the acquisition system, such as an Intan acquisition board or the Open Ephys device (Siegle et al., 2017). For this standard mapping, we can directly use a ProbeInterface pathway.


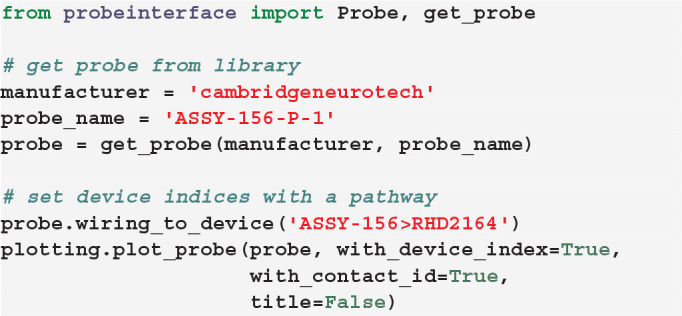


**Figure 4:**
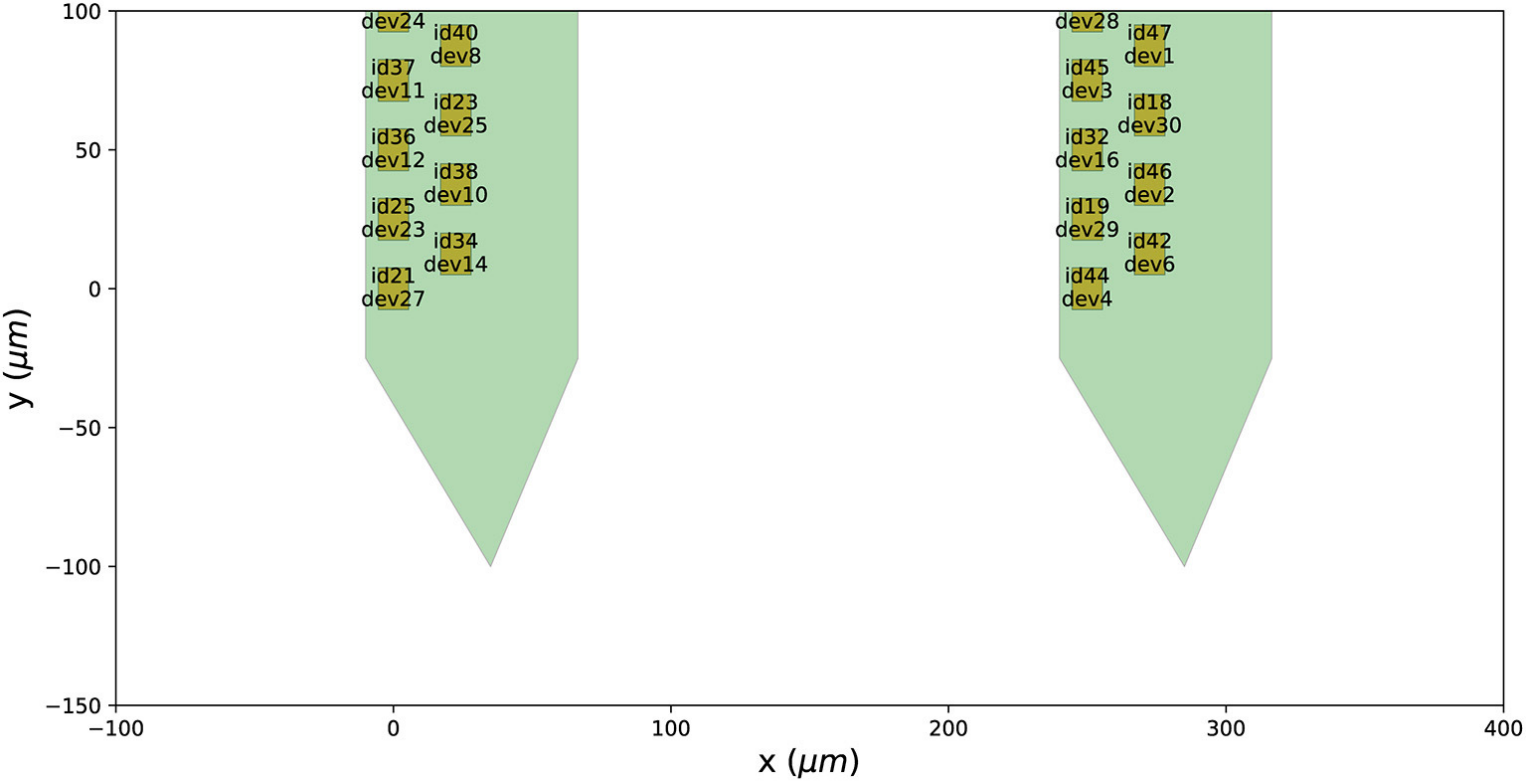
**Wiring a probe to an acquisition device**. When a probe is *wired* to a device, each contact is assigned a device_channel_index, which indicates to which recorded trace it corresponds to. For each contact, the text shows both the contact id (e.g., *id21*) and the device channel index (e.g., *dev27*).

After mapping the probe, each contact is assigned a device_channel_index which uniquely associates the channel to the recorded traces. In Figure 4, we show a zoomed in view of the bottom part of the first two shanks of the probe, with each contact labeled with its contact id and device channel index. The bottom left channel of the first shank (contact id 21) is now wired to the 28th recorded signal (0-index). Clearly, this information is very relevant for every downstream analysis. Note that probe groups can be wired similarly using the set_global_device_channel_indices() function.

As new probes, headstages, and connectors are developed and adopted by the community, the number of available *pathways* in ProbeInterface will grow. We encourage the neuroscience community to contribute to this open-source project and validate existing and add new pathways as they become available.

## 4. File Format and I/O

While there are some existing file formats to describe neural probes for data analysis, mainly related to spike sorting, these are limited to the minimal information needed for signal processing and are therefore incomplete. As an example, the .prb file, used by Klusta (Rossant et al., 2016) and Spyking-CIRCUS (Yger et al., 2018) to provide probe information for the spike sorting pipeline, only contains information about the locations of the contacts and their *channel group*. While these two pieces of information are probably enough for spike sorting, additional characteristics are relevant for other applications. For example, when computing extracellular signals from biophysically detailed neuronal models, in order to model the spatial extent of each recording site, information about the shape and the size of the electrodes is also required (Hagen et al., 2018; Buccino and Einevoll, 2020).

Given these limitations, we therefore introduce a flexible description based on the established JSON format. The ProbeInterface description of a *Probe* is an attempt to include all relevant information about the probe(s) used in an experiment. The JSON implementation allows easy access by existing frameworks in different programming languages, from spike sorting tools to visualization and modeling packages. ProbeInterface includes multiple features to ensure its long-term usability and flexibility.

First, a ProbeInterface file includes the version of the ProbeInterface package, to ensure reproducibility of the analysis.


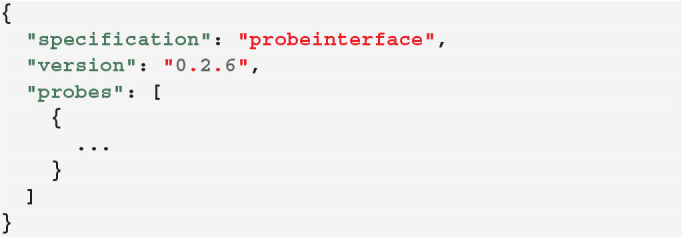


Second, the probes field allows to specify multiple probes in the same file (i.e. a probe group). This feature allows to describe multiple probes recorded with the same acquisition device, as modern acquisition systems support a growing number of probes. Each entry of the probes list must contain the following required fields:

ndim: number of dimensions (2 or 3)
si_units: units for the positions (um, mm, etc.)
annotations: free-text field. It can contain the *name* of the probe, the *manufacturer*, a *description*, and more
contact_positions: list of 2D or 3D positions (depending on the ndim of the probe contacts)
contact_shapes: shapes of the electrodes. It can be circle, square, or rect. This field can be either a single string (in this case all contacts share the same shape) or a list of shapes with the same length as the number of contacts (this allows one to describe probes with different electrode shapes)
contact_shape_params: this field specifies parameters for the contact shapes. Similarly to contact_shapes, it can be a single dictionary (all contacts have the same shape) or a list of dictionaries with the same length of the number of contacts. The shape parameters are radius for the circle, width for the square, and width and height for the rect shapes.

In addition to these mandatory fields, other fields can be optionally specified. These include:

contact_plane_axes: orientation of the contacts. This field gives the possibility to describe the orientation of each contact. For example, a square or a rectangular contact can be rotated with respect to the probe. The contact_plane_axes represents the 2D or 3D axes (depending on ndim) parallel to the sides of the contact (note that this only holds for rectangular and square contacts, given the symmetry of circular sites).
probe_planar_contour: this field allows to specify the contour of the probe. It contains a list of 2D or 3D points (depending on ndim) that describe the vertices of the probe contour.
device_channel_indices: when the probe(s) are wired to a device, this field contains the device indices.
shank_ids: this field contains the shank index for each contact (in case of a multi-shank probe, such as the one shown in Figure 3 - right).

ProbeInterface also implements an I/O module to read from and write to other file formats that describe probes. Several newly developed configurable probes, e.g. Neuropixels (Jun et al., 2017), can be set to record from different probe configurations and the output file [for Neuropixels a SpikeGLX format (Karsh)] contains this information. In this case, a probe object can be loaded directly from the acquired file:


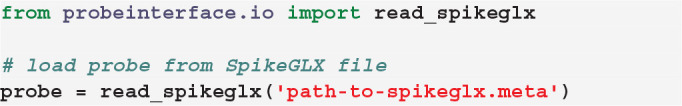


Similarly, ProbeInterface can automatically load a probe object from Maxwell Biosystems files^1^, a MEArec simulator file (Buccino and Einevoll, 2020), a .prb file, a Neurodata Without Borders (NWB) file (Teeters et al., 2015; Rübel et al., 2021), and an electrophysiology Brain Imaging Data Structure (BIDS) file (Gorgolewski et al., 2016).

In addition, each Probe or ProbeGroup object can also be written to a ProbeInterface file, a .prb file, and an ephys BIDS file set.

## 5. Integration With SpikeInterface

SpikeInterface^2^ is an open-source framework to unify extracellular electrophysiology analysis and spike sorting (Buccino et al., 2020). Since version 0.90, ProbeInterface is integrated into SpikeInterface, and this allows users to directly attach a Probe or ProbeGroup object to a SpikeInterface recording object. Internally, the ProbeInterface layer is used to provide spike sorters with the necessary probe information for sorting and for visualization purposes. This example shows how to register a Probe object into SpikeInterface and to run a spike sorter, e.g., Tridesclous (Garcia and Pouzat, 2015):


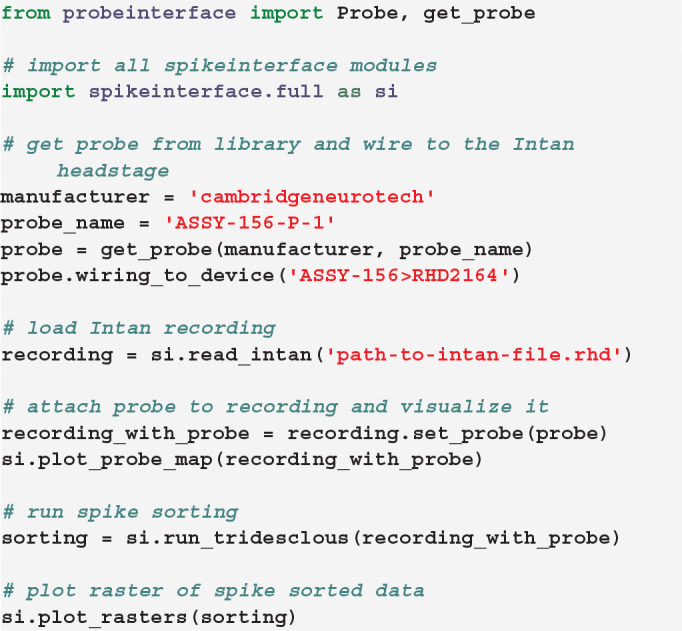


If the Probe object contains multiple shanks or a ProbeGroup is used instead of a Probe object, the user can optionally choose to use this information to assign channels to different *groups* by using the by_shank=True or the by_probe=True options of the set_probe() function, respectively. This enables spike sorting to be performed separately for each group, as spikes from the same neuron are not expected to appear on different shanks or probes.

## 6. Discussion

We present ProbeInterface, a lightweight and user-friendly Python framework to standardize the description and handling of neural probe mapping for subsequent data analysis. After introducing the main concepts of the ProbeInterface framework (Figure 1), we demonstrate how the Python API can be used to create a probe configuration from scratch (Figure 2) and to retrieve a commercial probe from the ProbeInterface public library (Figure 3). Finally, we describe the file format used by ProbeInterface and how it can interface with other available file formats.

Probe description and handling in neuroscientific experiments is far from standardized. The plethora of available probes, connectors, headstages, and acquisition system makes each experimental setup unique. Arising from this, current analysis frameworks mainly start from raw signals, taking the steps that go from the probe to the acquisition system for granted. With ProbeInterface we aim to improve standardization and reproducibility starting from the underlying signal pathway from probe to computer. By providing an enhanced description of neural probes and automatic mapping between contact and device indices we facilitate the use of this important information by analysis tools. In fact, aspects of the probe design, e.g., the size and shape of the electrodes, the material, the impedance and coating, arguably influence the quality of the signals and it is, therefore, important to track and include this information in the processing pipeline and data sharing. By collaborating with Cambridge Neurotech, one of the major vendors of neural probes for extracellular electrophysiology, we provide a readily available and validated library of over 100 neural probe designs. We envision that other companies and research groups developing probes will contribute to this open library in the near future.

The information about the probe configuration is essential for spike sorting, which is a required and delicate processing step to extract single-neuron activity from extracellular signals. In this regard, ProbeInterface can facilitate a standardized description of probe information among available sorters, which currently require tool-specific files and configurations to describe the neural probes. ProbeInterface is already integrated in SpikeInterface (Buccino et al., 2020) (version>0.90), allowing to directly load a ProbeInterface object into a processing pipeline. The integration with SpikeInterface, which internally supports over 10 spike sorting frameworks, is a first important step in the direction of standardizing probe handling in neuroscience and we foresee that other analysis tools will adopt ProbeInterface to describe and handle probe information.

Finally, in addition to standardization of probe handling for analysis pipelines, we are in the process of integrating ProbeInterface objects as extensions to standard file formats employed across the neuroscience community, including Neurodata Without Borders (NWB) (Teeters et al., 2015; Rübel et al., 2021) and Brain Imaging Data Structure (BIDS) (Gorgolewski et al., 2016) formats. Standardized data formats in neuroscience are arguably improving reproducibility and facilitating data sharing and ProbeInterface integration contributes an user-friendly and comprehensive way to add probe information to these files.

## Data Availability Statement

Publicly available datasets were analyzed in this study. This data can be found at: https://github.com/SpikeInterface/probeinterface.

## Author Contributions

SG and AB conceptualized the design and the project. SG, JS, and AB developed the software. TH contributed to the probe library. AB wrote the initial draft of the manuscript. SG, JS, TH, and AB revised and approved the final version of the manuscript. All authors contributed to the article and approved the submitted version.

## Funding

This work was supported by the ETH Zurich Postdoctoral Fellowship 19-2 FEL-17 (AB) and by the French Agence Nationale de la Recherche, under project 19-DATA-0021-01 (JS).

## Conflict of Interest

TH is the founder and CEO of Cambridge Neurotech. The remaining authors declare that the research was conducted in the absence of any commercial or financial relationships that could be construed as a potential conflict of interest.

## Publisher's Note

All claims expressed in this article are solely those of the authors and do not necessarily represent those of their affiliated organizations, or those of the publisher, the editors and the reviewers. Any product that may be evaluated in this article, or claim that may be made by its manufacturer, is not guaranteed or endorsed by the publisher.

## Footnotes

^1^*MaxWell Biosystems*. Available online at: https://www.mxwbio.com/

^2^ https://spikeinterface.readthedocs.io/en/latest/
